# Thriving in Youth with Autism Spectrum Disorder and Intellectual Disability

**DOI:** 10.1007/s10803-015-2412-y

**Published:** 2015-03-15

**Authors:** Jonathan A. Weiss, Priscilla Burnham Riosa

**Affiliations:** Department of Psychology, York University, Behavioural Science Building, 4700 Keele Street, Toronto, ON M3J 1P3 Canada

**Keywords:** Autism spectrum disorder, Intellectual disability, Special Olympics, Thriving, Mental health, Positive psychology, Positive outcomes

## Abstract

Most research on mental health in individuals with autism spectrum disorder (ASD) and intellectual disability (ID) has focused on deficits. We examined individual (i.e., sociocommunicative skills, adaptive behavior, functional cognitive skills) and contextual (i.e., home, school, and community participation) correlates of thriving in 330 youth with ID and ASD compared to youth with ID only, 11–22 years of age (*M* = 16.74, *SD* = 2.95). Youth with ASD and ID were reported to thrive less than peers with ID only. Group differences in sociocommunicative ability and school participation mediated the relationship between ASD and less thriving. Research is needed to further elucidate a developmental-contextual framework that can inform interventions to promote mental health and wellness in individuals with ASD and ID.

## Introduction

Individuals with autism spectrum disorder (ASD) and intellectual disability (ID) have significant and pervasive support needs across many life domains, including educational, health, and community areas, and many struggle with emotional and behavior problems (Mannion et al. 2014; Simonoff et al. 2008; White et al. 2009). In the most recent CDC (2014) report, 31 % of youth with ASD had intellectual skills in the ID range (with another 23 % in the borderline range), although estimates across studies range widely, from 26 to 68 % (CDC 2012; Fombonne 2005; Yeargin-Allsopp et al. 2003). We also know a great deal about the correlates of these pervasive needs, at individual (e.g., age, sex, diagnosis: Anagnostou et al. 2014), family (e.g., parent stress: Witwer and Lecavalier 2008), and more distal social levels (e.g., socio-economic status: Emerson and Hatton 2007). Understandably, research has largely focused on these problem behaviors and the remediation of negative outcomes, and we know far less about these youths’ strengths or how to promote positive outcomes, such as happiness, satisfaction, or resilience (Dykens 2006).

There is a role for positive psychology in identifying the characteristics of wellbeing and the situations that promote thriving, in a way that is more balanced than focusing solely on what is deficient (Gillham and Seligman 1999; Schalock 2004). Studies of positive or optimal outcomes of individuals with ASD are limited (Fein et al. 2013; Magiati et al. 2014). Indeed, thriving is an important but almost altogether unused term in the ASD research literature. Benson and Scales (2009) define thriving as “an individual’s pursuing a life path on which individual or functionally-valued behaviors grow (e.g., character, confidence, caring) and move the person toward attainment of an ‘idealized personhood’ characterized by socially or structurally-valued behaviors such as contribution to self, family, community, and civil society (Lerner 2006)” (p. 90). Thriving reflects both wellbeing and an upward developmental trajectory, the demonstration of continued growth of knowledge and skills, and success in relationships with others (Carver 1998), and ultimately, contributions in a meaningful way to oneself and one’s environments according to one’s potential (Hershberg et al. 2014). Thriving is thought to be the result of the “dynamic and bi-directional interplay over time” of an individual’s strengths and contexts (people, places) that support development (Benson and Scales 2009, p .90).

Positive youth development, and more broadly positive human development, has emerged as a promising framework with which to study thriving (Lerner et al. 20005a, b, 2010, 2011). Founded in relational systems theory, the positive youth development perspective posits that positive characteristics develop through mutually beneficial “*individual*-*context relations”* (Lerner 2005, p. 18), known as adaptive developmental regulations (Brandtstädter 1998). With a strong fit between an individual’s strengths (i.e., functional cognitive and behavioral skills) and their ecological resources (at the level of the home, school, and community), youth are more likely to show characteristics of thriving (Bowers et al. 2014; Lerner et al. 2010), often operationalized as the “6 Cs”: *Competence* (i.e., holding a positive view of one’s actions within social, academic, cognitive, and vocational domains), *Confidence* (i.e., an overall sense of self-worth and self-efficacy), *Character* (i.e., respect for societal and cultural rules, integrity), *Caring* or *Compassion* (i.e., sympathy and empathy), *Connection* (i.e., positive reciprocal bonds with people and institutions), and ultimately, *Contribution* (i.e., helping family, community, broader society, and self).

To date, no studies have empirically examined predictors of thriving in youth with ASD and ID. In typically developing children and adolescents, thriving is positively related to the degree of school and extra-curricular involvement (Agans et al. 2014; Bundick 2011), and positive parental attitudes (Callina et al. 2014), and is inversely related to maladaptive behavior (Arbeit et al. 2014; Geldhof et al. 2014; Lösel and Farrington 2012). Given that individual and contextual factors are related to self-determination in youth with ID,^1^ and the conceptual similarities between it and thriving, it may be that level of intellectual functioning (Wehmeyer et al. 2012), social skills (Carter et al. 2006), and adaptive coping skills (Fullerton and Coyne 1999) are relevant individual-level variables for youth with ASD and ID. Similarly, level of involvement in school (Shogren et al. 2013) and successful involvement in extra-curricular activities (Wehmeyer et al. 2010), may be important.

Studying the factors that explain thriving could lead to novel interventions that promote mental health and wellness, and complement the existing literature on interventions that focus on alleviating problems. This may be particularly important for youth with ASD and ID, who may be at risk of lower levels of thriving compared to peers with ID only. Individuals with ASD are known to have more difficulties in sociocommunicative functioning (Shattuck et al. 2011), have greater levels of associated psychiatric issues (Bradley et al. 2004; Brereton et al. 2006; Totsika et al. 2011), and have more difficulty engaging in school (Ashburner et al. 2010) and community activities (Orsmond et al. 2013; Shattuck et al. 2011; Solish et al. 2010) compared to peers of similar intellectual levels.

In the current study we compared levels of parent reported thriving in individuals with ASD and ID to those without ASD (ID only) and sought to determine the individual and contextual variables that predict this outcome. We hypothesized that youth and young adults with ASD and ID would achieve less thriving than peers with ID only. Further, we expected that individual (i.e., sociocommunicative skills, adaptive behavior, functional cognitive skills) and contextual factors (i.e., successful involvement in home, school, and community activities) would be related to thriving, and that group differences in these factors would address why individuals with ASD and ID would thrive less than youth with ID only. Such mediation would occur if the variance accounted for by the relation between ASD status and thriving were to be accounted for by the intermediate individual and contextual variables (Baron and Kenny 1986; Hayes 2012).

## Method

### Participants

The sample consisted of 330 family caregivers of youth and young adults registered with a community Special Olympics program in Ontario (Canada), between 11 and 22 years of age (*M* = 16.74; *SD* = 2.95), with 62 % of youth being male. To be included in the study, all individuals received a clinical diagnosis of ID by a registered health professional, verified through parent report of intellectual functioning and report of etiology. Although we cannot ensure the diagnostic status of participants beyond parent report, similar processes have been used to ascertain developmental disability in large-scale parent report surveys of youth with ASD and ID (Daniels et al. 2011; Kogan et al. 2008, 2009; Lin et al. 2012; Totsika et al. 2011), and to be eligible to participate in Special Olympics, caregivers indicate that individuals have an ID at the point of registration. Further, Special Olympics Ontario (SOO) is described as a sport organization for individuals with ID, and caregivers indicated that their children had ID at the point of registration, after reading the following definition of ID:

Persons with an ID are eligible to participate in Special Olympics. A person is considered to have an IQ if that person satisfies the following requirements: (1) Typically an IQ score of approximately 70 or below; (2) Deficits in general mental abilities which limit and restrict participation and performance in one or more aspects of daily life such as communication, social participation, functioning at school or work, or personal independence, and; (3) Onset during the developmental period (before the age of 18 years). All individuals 8 years of age or older, who have an intellectual disability have access to SOO sport programs. Individuals who have multiple disabilities are also eligible to participate so long as one of the disabilities is an intellectual disability.

Approximately 29 % of the sample was reported to also have a diagnosed ASD. Table 1 provides details on demographic characteristics of the overall sample and of the groups. When comparing youth with ID and ASD to those with ID only, the only significant difference was with respect to child sex, with a greater proportion of males in the group with ID and ASD (78 vs. 55 %).

**Table 1:** Participant demographics

	Overall *N* = 330 *M* (*SD*) or *N* (%)	ID only *n* = 235 *M* (*SD*) or *N* (%)	ID and ASD *n* = 95 *M* (*SD*) or *N* (%)	
Child age	16.78 (2.92)	16.93 (2.82)	16.40 (3.12)	*t*(328) = 1.50, *p* = .13, *d* = .17
Child gender (% male)	203 (62 %)	129 (55 %)	74 (78 %)	*Χ* ^2^ (1) = 15.1 *p* < .001, *Cramer’s* *V* = *.21*
*Level of competition*				
Local	267 (82 %)	191 (82 %)	76 (82 %)	
Provincial	48 (15 %)	35 (15 %)	13 (14 %)	*Χ* ^2^ (2) = .38, *p* = .83,
National/International	11 (3 %)	7 (3 %)	4 (4 %)	*Cramer’s* *V* = *.03*
*Training in the last year*				
None or a few times	84 (26 %)	61 (26 %)	23 (25 %)	
1–4 times per month	149 (45 %)	102 (43 %)	47 (51 %)	*Χ* ^2^ (2) = 1.59, *p* = .45,
At least twice per week	95 (29 %)	72 (31 %)	23 (25 %)	*Cramer’s* *V* = .07
Total sports in 12 months	2.3 (1.5)	2.3 (1.5)	2.3 (1.4)	*t*(313) = .52, *p* = .60
Respondent source (% mothers)	268 (82 %)	195 (83 %)	73 (77 %)	*Χ* ^2^ (1) = 1.89, *p* = .17, *Cramer’s* *V* = .07
*Geographical location*				
Remote	11(3 %)	9 (4 %)	2 (2 %)	*Χ* ^2^ (3) = 1.09, *p* = .78,
Rural	82 (25 %)	56 (24 %)	26 (28 %)	*Cramer’s* *V* = .06
Suburban	141 (44 %)	100 (44 %)	41 (44 %)	
Urban	89 (28 %)	65 (28 %)	24 (26 %)	
*Respondent educational level*				
High school or less	63 (19 %)	46 (20 %)	17 (18 %)	
College degree or equivalent	121 (37 %)	87 (37 %)	34 (36 %)	*Χ* ^2^ (2) = .34, *p* = .84,
University degree	144 (44 %)	100 (43 %)	44 (46 %)	*Cramer’s* *V* = .03
*Finances before taxes*				
<49,000	48 (18 %)	35 (18 %)	13 (17 %)	
50,000–99,999	117 (43 %)	79 (41 %)	38 (48 %)	
100,000–149,999	82 (30 %)	60 (31 %)	22 (28 %)	*Χ* ^2^ (3) = 1.57, *p* = .67,
150,000 or greater	27 (10 %)	21 (11 %)	6 (8 %)	*Cramer’s* *V* = .08
*Financial management*				
Doing well	137 (43 %)	102 (45 %)	35 (37 %)	
Get by alright	131 (41 %)	90 (40 %)	41 (44 %)	*Χ* ^2^ (2) = 1.61, *p* = .45,
Financial trouble	54 (17 %)	36 (16 %)	18 (19 %)	*Cramer’s* *V* = .07
Family difficulties	3.26 (.48)	3.29 (.49)	3.21 (.47)	t(327) = 1.79, *p* = .08, *d* = .20

Most youth (93 %) lived with at least one of their parents with 15 % of youth living in a single-parent household. Mothers were the most common respondents in the survey (81 %), followed by fathers (13 %). Most respondents were married (83 %). Respondent educational attainment was as follows: High school degree or less (19 %), college/trade/non-university diploma (37 %), university degree (44 %). Fifty-six percent of parents reported a total before-tax household income under $100,000 (CAD) per year (median 2012 provincial household income = $74,890 CAD; Statistics Canada 2014). Financial status was also evaluated using a single question in which parents were asked how well they were managing (National Centre for Social Research and Department for Work and Pensions 2011; 1 = *managing well* to 7 = *deep financial struggle*; see also Emerson and Hatton 2007), with 17 % reporting some degree of financial struggle. Respondents reported living in rural or remote (29 %), suburban (44 %), and urban areas (28 %). Caregivers also reported on the overall functioning of the family using the 12-item General Functioning Scale of the McMaster Family Assessment Device (Epstein et al. 1983), with no difference between the group with ID and ASD and those with ID only.

#### Recruitment

All participants were sampled from SOO (Canada) registration lists. Special Olympics is the largest community sport organization for people with developmental disabilities in the world, found in over 170 countries, with over 4.4 million registrants (Special Olympics 2015a). Special Olympics currently has 544,581 registered athletes in North America (Special Olympics 2015b). Although some studies have examined athletes who participate at high-level competitive events (Dykens and Cohen 1996), Special Olympics is primarily a grassroots community-based organization with the goal of promoting community participation, health, and wellbeing of individuals with developmental disabilities through sport.

The degree of involvement in Special Olympics varied considerably, suggesting that this sample did not reflect an intensely involved or elite group of athletes. Most youth competed in Special Olympics only at local levels (82 %). In the last year, 24 % of the sample participated in no or only a few training sessions with Special Olympics, with another 46 % training between one and four times in a month, and 29 % participating at least twice per week. Of those who participated at least a few times in the last year, it was on average in two sports (*SD* = 1.5), with the mode being one sport (33 %). There were no caregiver or Special Olympics differences between youth with ID and ASD and those with ID only.

### Measures

#### Adaptive Behavior

The Waisman Activities of Daily Living Scale (W-ADL; Maenner et al. 2013) was used as a measure of adaptive behavior. The W-ADL is a 17-item 3-point scale that is used to measure an individual’s independence in doing a variety of activities of daily living, such as ‘making his/her own bed’ and ‘drinking from a cup’ (0 = *does not do at all*, 1 = *does with help*, 2 = *independent or does on own*). Total scores may range from 0 to 34 (current sample: range = 0–34, Median = 21.0, *M* = 20.70, *SD* = 6.31). The W-ADL was developed and validated for use with parents of adolescents and adults with ASD and with ID (12–48 years of age), has demonstrated criterion and construct validity, including high correlations with the Vineland Adaptive Behaviour Scale Composite Score and Daily Living subscale score (*r* = 0.78 and *r* = 0.82, respectively; Maenner et al. 2013). It has high internal consistency across samples with different disabilities (Cronbach’s α = 0.88–0.94; Maenner et al. 2013), which was equally high in the current study (Cronbach’s α = .91). The W-ADL has been used in other studies with adolescents and adults with ASD (Smith et al. 2012; Taylor et al. 2014).

#### Sociocommunicative Ability

Sociocommunicative ability was measured by combining a set of items measuring social and communicative functioning. Social abilities were measured through a brief social scale used in other research with parents of adolescents and adults with ASD (Anderson et al. 2014; Frazier et al. 2011; Mazurek et al. 2012; Sterzing et al. 2012; Wei et al. 2014a; b), taken from the National Longitudinal Transition Study—2 (NLTS2), a nationally representative study of adolescents receiving special education services in the U.S. The 4-item 4-point scale is used to ask parents how often their child joins groups without being told to; makes friends easily; seems confident in social situations; and starts conversations rather than waiting for others to initiate (1 = *never*, 2 = *sometimes*, 3 = *often*, 4 = *very often*). Previous use of these items with parents of individuals with ASD indicated good internal consistency (Mazurek et al. 2012; Cronbach’s α = .75), which was better in the current study (Cronbach’s α = .85). With regard to communication, we developed a 3-item 4-point scale in which parents reported on how well their child understands spoken language, uses spoken language to communicate, and carries on a conversation, based on the single item scale by Mazurek et al. (2012) and Sterzing et al. (2012) (1 = *cannot do this at all*, 2 = *has a lot of trouble*, 3 = *has a little trouble*, 4 = *has no trouble*). The scale had good internal consistency (Cronbach’s α = .83). We combined the social and communicative items to better reflect current conceptualizations of social communication impairments in ASD as one set of criteria, and that in the current sample, the two scales were moderately correlated (*r* = .50, *p* < .001). Mean scores were taken across the seven items, with higher scores reflecting greater sociocommunicative competence, with possible scores ranging from 1 to 4 (current sample: range of mean scores = 1.29–4, Median = 2.71, *M* = 2.71, *SD* = .63). The overall scale had good internal consistency with the current sample (Cronbach’s α = .82) and a moderate interclass correlation (single measures = .39, average measures = .82).

#### Functional Cognitive Ability

Functional cognitive abilities were measured on a 4-item 4-point scale used in previous research to measure functional cognitive abilities through parent reports in adolescents and adults with ASD (Frazier et al. 2011; Mazurek et al. 2012; Shattuck et al. 2012; Sterzing, et al. 2012), and originally used in the NLTS2. Parents were asked how well the child tells time on an analog clock, reads and understands common signs, counts change, looks up telephone numbers, and uses a telephone (0 = *not at all well*, 1 = *not very well*, 2 = *pretty well*, 3 = *very well*). Scores range from 0 to 3 with higher scores indicating better functional cognitive abilities (current sample: range of mean scores = 0–3, Median = 1.25, *M* = 1.34, *SD* = .81). The current study’s sample had good internal consistency (Cronbach’s α = .84), similar to past research with this measure (Cronbach’s α = .85; Mazurek et al. 2012). Functional cognitive ability was correlated with composite IQ scores obtained on a subsample of participants,^2^ *r*(49) = .58, *p* < .001, using the Wechsler Abbreviated Scales of Intelligence (WASI:2; Wechsler 2011). This measure of functional skills was not meant to be a proxy for IQ, as the two are distinct constructs (Roux et al. 2013).

#### Involvement in Home, School, and Community

Involvement in external environments was rated by parent report on the Participation and Environment Measure (PEM-CY; Coster et al. 2012). Coster et al. (2012) developed this measure to assess the frequency of participation (*daily* to *never*) and level of involvement (*very involved* to *minimally involved*) of children and adolescents with physical and cognitive disabilities (including children with ASD and ID in their validation sample). In the current study, we examined the overall mean frequency of participation in home (10 items; e.g., ‘homework’, ‘watching tv’), school (5-items; e.g., ‘field trips and school events’, ‘special roles at school’), and community (10 items; e.g. ‘neighborhood outings’, ‘community events’) domains. Frequency of participation was rated on an 8-point scale (1 = *daily*, 2 = *few times a week*, 3 = *once a week*, 4 = *few times a month*, 5 = *once a month*, 6 = *few times in last* 4 *months*, 7 = *once in last* 4 *months*, 8 = *never*), and scores are reverse coded (8 = 0 to 1 = 7) so that higher scores indicated greater participation (ranging from 0 = *never* and 7 = *daily*). Mean scores were calculated for each domain. For the community domain, actual mean scores ranged from .20 to 6.10 (*M* = 3.25, Median = 3.2, *SD* = 1.04). For the home domain, actual mean scores ranged from 2.5 to 7.0 (*M* = 5.52, Median = 5.7, *SD* = .90). For the school domain, actual mean scores ranged from .20 to 6.60 (*M* = 3.13, Median = 3.2, *SD* = 1.34). The initial validation study reports acceptable to good internal consistency across home (Cronbach’s α = .59), school (Cronbach’s α = 61), and community (Cronbach’s α = .70), with similar rates in the current study (α = .56–.76).

#### Thriving

Thriving was measured using a parent report scale of the six Cs of positive youth development, derived from the 4-H study, an 8-wave longitudinal investigation involving over 7,000 youth in the U.S (Lerner et al. 2005a). This parent report measure was designed to assess the youth’s competence, confidence, character, connection, caring, and contribution and it has been used with over 4,000 parents of youth in the 4-H Study (Lerner et al. 2005a). Characteristics of thriving are meant to be global statements about positive youth development, rather than specific elements related to one or two domains. Parents were asked to rate their level of agreement to a global statement about each of the six characteristics on a 5-point scale (1 = *strongly disagree*, 2 = *disagree*, 3 = *neither agree nor disagree*, 4 = *agree*, 5 = *strongly agree*). The six items are listed in Table 2. A mean score was calculated across all items, with strong internal consistency (Cronbach’s α = .85), and a moderate interclass correlation (single measures = .49, average measures = .85). Actual mean scores ranged from 1.17 to 5 (*M* = 3.71, Median = 3.83, *SD* = .80).

**Table 2:** Item description for each thriving component, percentage of parent agreement, and group comparisons

	ID only Mean rank; *M* (*SD*)	ID and ASD Mean rank; *M* (*SD*)	Mann–Whitney U *z*-score
Competence: my child has the skills to succeed in school, in social situations with friends and adults, in play, and at home. My child knows how to behave and does what is needed to do well	175.02; 3.29(1.11)	140.32; 2.85(1.20)	−3.11, *p* = .002
Confidence: My child believes that he/she can succeed and do what is needed to do well in the family, in school, in social situations with friends and adults, in play and in other areas that are important to him/her (for example, sports, music, religious activities)	174.63; 3.56(1.02)	141.29; 3.14(1.18)	−3.03, *p* = .002
Connectedness: my child has positive relationships with his/her parents, siblings, and other family members, and with friends, teachers, coaches, or mentors	170.45; 4.35(0.81)	153.26; 4.18(0.91)	−1.63, *p* = .10
Character: my child knows what is right and wrong; and does the right thing; My child is open to others’ perspectives and believes in social justice for all. My child is honest	166.67; 3.69(1.07)	160.89; 3.62(1.10)	−.52, *p* = .60
Caring: my child cares about other people. He or she is concerned about whether others have what they need (shows sympathy) and shows a sense of compassion (empathy). My child is both sympathetic and empathetic to others	179.49; 4.19(0.95)	128.79; 3.56(1.20)	−4.64, *p* < .001
Contribution: my child tries to do things to help the family, to help neighbors, and to help the community. My child tries to also help himself/herself by staying healthy (eating right, exercising, getting enough sleep)	170.81; 3.76(1.10)	145.72; 3.46(1.14)	−2.28, *p* = .023

### Procedure

Family caregivers of every athlete in SOO, who was between 11 and 21 years of age in 2012 (*N* = 2800), were contacted via email and mail using a modified version of the Dillman recruitment method (Dillman 2000), and invited to participate in an online or paper-and-pencil survey about involvement in Special Olympics. Data collection occurred from April to September 2013. Ethical approval was obtained from York University and SOO and informed consent was obtained from all participants. Our original sample represented 19 % of all registered athletes (*N* = 434) in this age range, although not all participants completed all the measures of interest. A comparison with the overall registration dataset revealed that participants did not differ from non-participants in athlete age, gender, or geographic distribution (all *p* > .05).

### Data Analytic Procedure

All statistical analyses were performed using IBM SPSS version 21. Mann–Whitney U and *t* tests were used to test the hypothesis that youth with ID and ASD would show less thriving compared to youth with ID only, and to examine any differences in the individual and context variables. We tested the possibility of multiple mediators using the PROCESS macro (Hayes 2012), which is advantageous over traditional regression techniques (Baron and Kenny 1986) as it can compute mediator paths after controlling for the variance associated with competing mediators (i.e., the shared variance), providing greater independence among the variables. For the current analysis, we selected PROCESS Model 4, designed specifically for multiple mediation. Given the limited sample size, and to prevent violation of normal distribution, 1000 bootstrap samples were drawn as a robust estimation of direct and indirect effects (Farmer 2012; Preacher and Hayes 2008). Bootstrapping provided a confidence interval (CI) around the indirect effects. Mediating factors were considered significant if the intervals between the lower and upper limit of a 95 % CI did not contain zero (Preacher and Hayes 2008). The PROCESS macro allows for an exploration of multiple simultaneous mediation as well as conventional direct multiple regression to assess how each variable is related to thriving after controlling for the variance accounted for by the other variables of interest.

All variables had skewness and kurtosis estimates within acceptable limits, and no major violations of distributions were noted upon visual inspection of histograms and Normal Q–Q Plots. Further, the bootstrap CIs provide a robust estimate in the face of non-normal distributions (Hayes 2015). As shown in Table 3, none of the predictor variables were correlated with each other above *r* = .54, and most were of small to moderate size. Standard regression collinearity (VIF estimates) and multicollinearity diagnostics (Condition Index/Variance Proportions) revealed no evidence of collinearity.

**Table 3:** Correlations among individual, contextual, and outcome variables

	Adaptive behavior	Sociocommunicative skills	Functional cognitive skills	Community participation	Home participation	School participation
Sociocommunicative skills	.35**	–				
Functional cognitive skills	.53**	.39**	–			
Community participation	.25**	.19**	.14*	–		
Home participation	.41**	.33**	.29**	.37**	–	
School participation	.21**	.34**	.12	.31**	.42**	–
Thriving	.33**	.54**	.32**	.31**	.38**	.43**

* *p* ≤ .01; ** *p* ≤ .001

## Results

### Differences in Internal Strengths and External Resources

As shown in Table 4, youth with ID and ASD and those with ID alone did not differ with respect to their levels of overall functional adaptive behavior nor did they differ with respect to their levels of functional cognitive skills. Youth with ID and ASD were reported to have significantly lower sociocommunicative abilities (with a large effect size, Cohen’s *d* = .79), compared to peers with ID only of the same age and level of adaptive functioning. Youth with ID and ASD were also rated to participate less in home and school activities than youth with ID only, with small to medium effect sizes, and marginal group differences in community participation.

**Table 4:** Individual and contextual variables in youth with ID only and youth with ASD and ID

	ID only *M* (*SD*)	ID and ASD *M* (*SD*)	
Adaptive behavior	20.86 (6.42)	20.62 (5.97)	*t*(328) = .32, *p* = .75, *d* = .04
Sociocommunicative skills	.2.86 (.59)	2.35 (.56)	*t*(328) = 7.16, *p* < .001, *d* = .79
Functional cognitive skills	1.31 (.78)	1.44 (.87)	*t*(328) = −1.38, *p* = .17, *d* = .16
Home participation	5.62 (.89)	5.26 (.90)	*t*(328) = 3.28, *p* = .001, *d* = .40
School participation	3.31 (1.30)	2.71 (1.35)	*t*(328) = 3.72, *p* < .001, *d* = .45
Community participation	3.32 (1.06)	3.08 (1.00)	*t*(328) = 1.90, *p* = .06, *d* = .23

### Differences in Thriving

Youth with ID and ASD were reported to have significantly less overall thriving than youth with ID only as well as in four specific elements of thriving. As shown in Table 2, Mann–Whitney *U* tests revealed that youth with ID and ASD were rated as having lower levels of competence, confidence, caring, and contribution compared to youth with ID only, with small to medium effect sizes between groups. An independent samples *t* test confirmed that youth with ID and ASD were rated lower on overall thriving (*M* = 3.47, *SD* = .82) than youth with ID only (*M* = 3.80, *SD* = .78; *t*(327) = 3.50, *p* < .001, *d* = .42).

### Mediators of Thriving

**Fig. 1:**
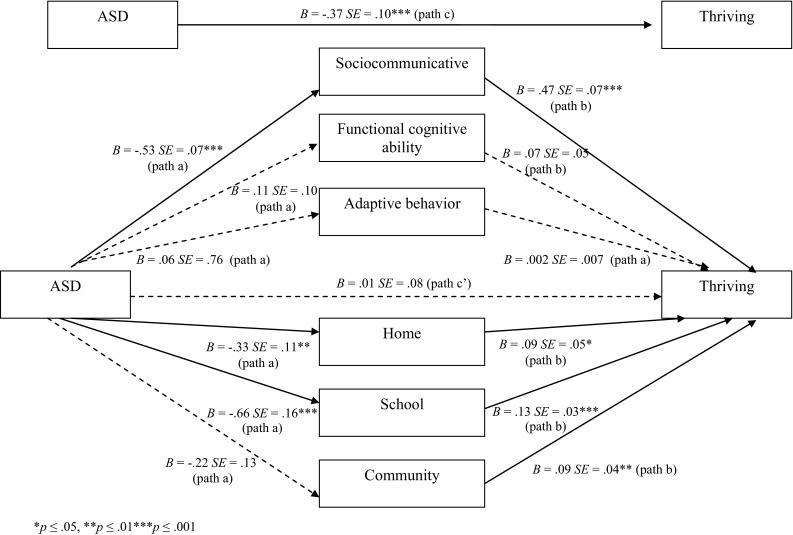
Multiple mediation model of thriving in youth with ID and ASD and ID only

Figure 1 displays the test of multiple mediation and the unstandardized coefficients of each pathway (PROCESS Model 4), after controlling for youth age and gender. The overall model of ASD status, control variables, and potential mediators accounted for 40 % of the variance in mean thriving, *F*(9, 318) = 23.77, *p* < .0001. As shown in Fig. 1 (path b), once all the variables were entered, sociocommunicative ability (*t* = 6.75, *p* < .0001), home (*t* = 1.90, *p* = .05), school (*t* = 4.21, *p* < .0001), and community (*t* = 2.47, *p* = .01) participation were all independent predictors of thriving.

As shown in Fig. 1 (path c), the total direct effect of ASD status was a significant predictor of thriving, prior to entering the mediator variables, *t* = −3.73*, p* = .0002, CI = −.55 to −.17. The multiple mediator results indicated that there was a significant total indirect effect for the set of six mediators (*point estimate* = −.38, CI = −.51 to −.23), and that this mediation was accounted for by the indirect effect of sociocommunicative skill (*point estimate* = −.25, CI = −.37 to −.15), participation at school activities (*point estimate* = −.08, −.15 to −.03), and to a lesser extent, at home (*point estimate* = −.03, CI = −.09 to −.004). The direction of estimates indicated that having ASD was related to less sociocommunicative skill and less participation at home and school (path a), which in turn were related to less thriving (path b). Further, the relation between ASD status and thriving was no longer significant after entering in the mediators (path c’), *t* = .11, *p* = .91, suggesting that these individual and contextual variables explained considerable variance associated with ASD status.

## Discussion

To our knowledge, this is the first study to examine thriving in youth with ID and ASD compared with youth with ID alone. As expected based on Lerner’s (2005) positive youth development framework, both individual and contextual variables were related to parent reported levels of thriving. By conceptualizing thriving as an individual-contextual process, we were able to in part explain why these youth with ASD and ID thrive less –because of differences in their level of sociocommunicative functioning and participation at home and school, relative to peers with ID only.

By definition, a diagnosis of ASD involves having impaired sociocommunicative functioning beyond what would be expected by an individual’s developmental level, so it is logical that the group with ASD and ID would have lower levels of sociocommunicative skill than those with only ID of similar functional cognitive and adaptive abilities. The current findings lend support to the importance of addressing the core symptoms of ASD through evidence-based treatments (Wong et al. 2013), in order to increase youth wellbeing. Improvements in sociocommunicative abilities in individuals with ASD have been linked to positive changes in an ability to learn (Hsiao et al. 2013), to make and maintain friendships (Bauminger and Kasari 2000; Daniel and Billingsley 2010; Rotheram-Fuller et al. 2010), to experience empathy (Baron-Cohen 2000), and be successful in school and in the community (Chiang et al. 2013); critical elements of thriving. Thriving, however, was not exclusively explained by individual characteristics.

Even though youth with and without ASD in the current sample were involved to the same degree in Special Olympics, it is striking that youth with ID and ASD were participating less in home, school, and community environments, and that even after controlling for sociocommunicative ability, participation in home and school were mediators of thriving. Previous research has shown that youth with ASD are prone to experience social exclusion (Symes and Humphrey 2010). Considered a fundamental right (United Nations 2006), inclusion involves meaningful self-determined and developmentally appropriate participation, and an experience of belonging (Cobigo et al. 2012). Our results suggest that interventions are needed to assist both the individual and their environments (Wehmeyer and Garner 2003; Wehmeyer and Shogren 2008). There is mounting research in support of interventions that can be used to foster socially inclusive opportunities (White et al. 2007) and friendships (Calder et al. 2013), and that promoting success in youth with ASD can come from positive practices that mobilize contextual supports (e.g., Humphrey and Symes 2010). For example, interventions that teach typically developing peers how to identify and interact with youth with social difficulties result in positive social experiences for youth with ASD (Banda et al. 2010; Chan et al. 2009; Harper et al. 2008; Kasari et al. 2012).

Interventions are similarly needed to support families of individuals with ASD in their aims of fostering meaningful home participation. An important first step is to further understand what predicts successful participation in home activities for youth with ASD (Poon 2011). Challenges with home participation may be related to the higher levels of restricted interests and behaviors (Gabriels et al. 2005; Matson et al. 2008; Rodgers et al. 2012), emotional and behavioral problems (Bodfish et al. 2000; Brereton et al. 2006), and parental stress and mental health problems (Ogston et al. 2011) found in individuals with ASD and their families compared to those with ID without ASD.

Past research on self-determination, which shares many qualities of thriving, has underscored how the combination of individual-level (e.g., level of disability) and systemic factors (e.g., school and community) is important for positive outcomes among individuals with and without disabilities (Nota et al. 2007; Pierson et al. 2008; Shogren 2013; Ullrich-French and Smith 2009). More specifically, Walker et al. (2011) suggest that social strengths, environmental supports, and social inclusion of individuals with disabilities in community settings mediate associations among personal characteristics (e.g., level of disability) and self-determination. Our results support this hypothesis, as most of our measures were social in nature (i.e., social and communication skills and participation).

There are a number of limitations to this research. Some of the measures were brief with less well-established psychometric properties; therefore the results are to be interpreted with caution. The results from our study were based solely on parent report, and further research is needed to include alternative data collection sources to reduce the impact of shared variance and to further assess the reliability of the constructs (Lerner 2005). Research on positive youth development and positive psychology in ASD and ID is still in its infancy, with no existing measures of direct observation or self-report. In addition, we used a general measure to index thriving, and it may be useful to examine the relationship between ASD, internal and external strengths, and specific aspects of positive youth development (i.e., competence, confidence, character, connection, compassion, and contribution). Because the current sample involved participants registered in Special Olympics, these findings may not be representative of those who are not involved with the organization. At the same time, we sampled individuals at local community-based levels, and sampling from such levels is being used to understand the predictors of health of individuals with ID (Adler et al. 2004; Harris et al. 2003; Hild et al. 2008; Reid et al. 2003; Turner et al. 2008; Woodhouse et al. 2004), and in the current study, was used to explore within-subject processes related to thriving.

Future work examining thriving and related constructs in populations with developmental disabilities is needed. Beyond the survey approach used in the current study, other ways to explore thriving include: face-to-face interviews with individuals with developmental disabilities and/or their family members, caregivers, or professionals; use of other existing self- or caregiver-reported measures of thriving, behavioural observation methods, photoelicitation techniques (using photographs as a primary data source to understand participant experiences; e.g., Wang et al. 2000) or other analytic or evocative qualitative methods such as autoethnography (the researcher details a personal, self-reflective, narrative to understand social phenomena; e.g., Ellis 2004). Where possible, methods in which multiple perspectives, particularly those of individuals with developmental disabilities, would be an important contribution to this growing literature.

Finally, data were cross sectional and the analyses correlational in nature. The cross-sectional design of this study limits the inferences that can be made about causal relations at play; however, our results contribute to the very limited existing evidence on what relates to positive outcomes in individuals with ASD. Further work is needed also to examine the predictors of thriving in youth with ASD who do not have ID. Studying thriving over time will be important in understanding the nature of positive developmental trajectories of youth with ASD and ID.

## Conclusions

Informed by positive psychology, we approached the current study by looking at strengths as they relate to thriving. As a group, individuals with ID and ASD were reported to thrive less than their peers with ID only; however, our results also highlight possible mechanisms into different ways of addressing this deficit. Thriving is related to skills, but not in isolation. It is better explained by skills in the context of home, school, and community inclusion. Positive youth development is said to occur when there is a proper interaction (or alignment) between internal strengths and the supports within one’s environments (Lerner 2006; Lerner et al. 2005b), and this study provides initial insight into the roles that these variables play to explain thriving in this population. Future research is needed to examine contextual factors such as family social support, connections with peers, community cohesion or acceptance, and socioeconomic status as they relate to thriving in youth with ASD.

## Footnotes

1 Defined as “acting as the primary causal agent in one’s life and making choices and decisions regarding one’s quality of life free from undue external influence or interference” (Wehmeyer 1996, p. 24).

2 Demographics of these 49 participants were similar to the larger sample with 51 % being male, 25 % having an ASD diagnosis, and the average age of 16.12 years (*SD* = 2.91, ranging from 11 to 22 years).
